# Bacterial networks in Atlantic salmon with Piscirickettsiosis

**DOI:** 10.1038/s41598-023-43345-x

**Published:** 2023-10-13

**Authors:** Yoandy Coca, Marcos Godoy, Juan Pablo Pontigo, Diego Caro, Vinicius Maracaja-Coutinho, Raúl Arias-Carrasco, Leonardo Rodríguez-Córdova, Marco Montes de Oca, César Sáez-Navarrete, Ian Burbulis

**Affiliations:** 1 Doctorado en Ciencias de la Ingeniería, Departamento de Ingeniería Química y Bioprocesos, Escuela de Ingeniería, Pontificia Universidad Católica de Chile, Avenida Vicuña Mackenna 4860, 7820436 Santiago, Región Metropolitana Chile; 2 Centro de Investigaciones Biológicas Aplicadas (CIBA), Avenida Lago Panguipulli 1390, Puerto Montt, Región de Los Lagos Chile; 3 Laboratorio Institucional, Facultad de Ciencias de la Naturaleza, Escuela de Medicina Veterinaria, Universidad San Sebastián, Sede Patagonia, Avenida Lago Panguipulli 1390, Puerto Montt, Región de Los Lagos Chile; 4 Centro de Modelamiento Molecular, Biofísica y Bioinformática (CM2B2), Facultad de Ciencias Químicas y Farmacéuticas, Universidad de Chile, Avenida Dr. Carlos Lorca Tobar 964, 8380494 Santiago, Región Metropolitana Chile; 5 Beagle Bioinformatics, Santiago, Región Metropolitana Chile; 6 Programa Institucional de Fomento a la Investigación, Desarrollo e Innovación (PIDi), Universidad Tecnológica Metropolitana, Avenida Dieciocho 161, 8330383 Santiago, Región Metropolitana Chile; 7 Facultad de Ingeniería, Escuela de Ingeniería, Universidad Santo Tomás, Avenida Ejército Libertador 146, Santiago, Región Metropolitana Chile; 8 Departamento de Ingeniería Química y Bioprocesos, Pontificia Universidad Católica de Chile, Avenida. Vicuña Mackenna 4860, 7820436 Santiago, Región Metropolitana Chile; 9 Centro de Investigación en Nanotecnología y Materiales Avanzados (CIEN-UC), Pontificia Universidad Católica de Chile, Avenida Vicuña Mackenna 4860, 7820436 Santiago, Región Metropolitana Chile; 10 Centro de Investigación Biomédica, Facultad de Medicina y Ciencia, Universidad San Sebastián, Sede Patagonia, Avenida Lago Panguipulli 1390, Puerto Montt, Región de Los Lagos Chile; 11 Unidad de Genómica Avanzada, Facultad de Ciencias Químicas y Farmacéuticas, Universidad de Chile, Avenida Dr. Carlos Lorca Tobar 964, 8380494 Santiago, Región Metropolitana Chile

**Keywords:** Metagenomics, Applied microbiology, Infectious-disease diagnostics

## Abstract

An unbalanced composition of gut microbiota in fish is hypothesized to play a role in promoting bacterial infections, but the synergistic or antagonistic interactions between bacterial groups in relation to fish health are not well understood. We report that pathogenic species in the *Piscirickettsia*, *Aeromonas*, *Renibacterium* and *Tenacibaculum* genera were all detected in the digesta and gut mucosa of healthy Atlantic salmon without clinical signs of disease. Although *Piscirickettsia salmonis* (and other pathogens) occurred in greater frequencies of fish with clinical Salmonid Rickettsial Septicemia (SRS), the relative abundance was about the same as that observed in healthy fish. Remarkably, the SRS-positive fish presented with a generalized mid-gut dysbiosis and positive growth associations between Piscirickettsiaceae and members of other taxonomic families containing known pathogens. The reconstruction of metabolic phenotypes based on the bacterial networks detected in the gut and mucosa indicated the synthesis of Gram-negative virulence factors such as colanic acid and *O*-antigen were over-represented in SRS positive fish. This evidence indicates that cooperative interactions between organisms of different taxonomic families within localized bacterial networks might promote an opportunity for *P. salmonis* to cause clinical SRS in the farm environment.

## Introduction

The relationships between environment, diet, microbiome and fish health are not fully understood but represent a critical body of knowledge that has global economic, ecological and food supply consequences. One link unifying all these factors are the bacterial communities living in various compartments of the fish, such as the gut and luminal mucosa. There is a reasonable belief that colonization of Atlantic salmon (*S. salar*) by bacteria from the pathogenic genus *Piscirickettsia* have negative effects on fish development, which impacts the size and time fish might be harvested. For many reasons, great resources are spent monitoring fish for the presence of various bacteria known to cause disease without a certain understanding of the significance of their presence or threshold of commensal occurrence. There is evidence that specific microorganisms can be beneficial to fish health by synthesizing essential nutrients that promote gut integrity^1,2^, consuming critical factors needed by pathogenic bacteria^3^, disruption of pathogen quorum sensing^4–7^ or competitively-excluding pathogenic bacteria from colonizing gut mucosa^8,9^ and lumen^6,9,10^. Bacterial pathogens are thought to help each other by producing toxins^5,6,11,12^ or metabolites^13^ that promote expression of virulence factors or create conditions that enhance disease. These observations support the idea that the community network of so-called ‘healthy’ bacterial symbionts breaks down and ‘pathogenic’ bacterial networks form in which specific species opportunistically proliferate to cause disease. The possibility that bacteria from different taxonomic families cooperate to reduce host defenses against disease has been challenging to directly test in Atlantic salmon because of variations in habitat and limited resources to control parameters that influence measurement outcomes. Whether networks of bacteria from different families cooperate to restructure the bacterial communities living in the gut of Atlantic salmon in such a manner that promotes opportunistic infections is not known.

The qualitative composition and quantitative abundance of detectable bacteria in the intestinal compartment of Atlantic salmon varies with life stage, diet, and environment^13,14^. These bacterial networks differ between marine and freshwater fish life-stages^15^. Bacterial networks also vary between individuals, across the length of the gastrointestinal tract, and between intestinal contents and mucosal surfaces^16–18^. These observations show there are likely many parameters influencing microbiome composition and the interrelationships between bacterial network composition and health outcome is not known. Although current knowledge of the entire gut microbiota in fish is limited^19^, it is clear that alterations in gut bacterial network profiles affect enzyme production^20^, nutrient digestion and nutrient utilization^21,22^. Gómez et al.^13^, Ringø et al.^23^, Desai et al.^24^, and Harvey et al.^25^ all reported that both dietary and environmental factors affect the composition and ratio of various gut bacterial species in salmonids even though initial bacterial colonization of the gastrointestinal tract begins shortly after hatching^13^.

The gut microbiota of Atlantic salmon is specific to its local environment during the first stages of the life cycle and changes as soon as smolts migrate from fresh to saltwater^16^. Fish intestines harbor a combination of resident (autochthonous) microbiota, attached to the intestinal mucosa, and non-resident (allochthonous) microbiota, comprised of microbes appearing transiently and associated with digesta^14,15,26,27^. Intestinal bacteria in Atlantic salmon primarily belong to the phylum Proteobacteria, Fusobacteria, Firmicutes, Bacteroidota, Actinobacteria, and Verrucomicrobia. The phyla Proteobacteria, Firmicutes, and Bacteroidota often comprise up to 90% of fish intestinal bacterial networks^17^. Other factors that affect the type and abundance of bacteria detectable in Atlantic salmon include diet, freshwater-to-saltwater transition^28^**,** the captive state^29^, environmental season^30^, and developmental stage^31^. It is essential to consider that temperature and nutrient availability also shape the microbial communities in seawater and sediments^31^. Fish farming increases local nutrient levels by the addition of fish feed and feces to the marine environment^32^, which is believed to influence the microbiome of both farmed fish and other wild fish in the local ecology. Klemetsen et al.^33^ confirmed these influences do affect intestinal bacterial communities of farmed salmon by measuring changes in the structure of bacterial networks in response to environmental changes, utilization of different feeds, use of closed or semi‐closed recirculation systems, and transfer to unprotected seawater environments. These findings established a link between increasing ocean temperatures and the growth of bacterial families that harm fish health. These external factors might influence gut bacterial communities in ways that confound the interpretation of measurement data. Experimentally controlling for these farming (e.g., diet, feeding with functional compounds, antibiotic treatments, age of smoltification, and health status) and environmental (e.g., biogeography, water salinity, and seasons) variables is needed to help compare healthy versus diseased states independent of such external influences.

Changes in bacterial community structure on the surface of the gut lumen are thought to be a critical step in bacterial pathogenesis. Microorganisms secrete compounds under certain circumstances that may inhibit the growth of other organisms. Classic examples of these interactions include the synthesis of antibiotics. So-called ‘health-associated’ bacterial networks may promote positive growth associations between members, such as in quorum sensing and biofilm formation. These positive growth relationships can prevent newly arriving microorganisms from colonizing a local community by sequestering nutrients and preventing attachment, a process known as competitive exclusion. Including lactobacilli in the diet of Atlantic salmon was reported to protect fish mucosa from colonization by pathogenic bacteria. The cooperative growth, or co-occurrence, of two or more microorganisms during disease indicates the possibility they may express complementary metabolic pathways that cooperatively interact to promote disease physiology. These observations invoke the idea that networks of bacteria might work as a team to express virulence factors under certain conditions to vie for control of a community. Many research findings reported to date have focused on how antibiotic treatments affected the gut microbiome of salmonids during defined treatment periods. However, these studies have not produced knowledge about how the microbiome of salmonids behaves during naturally occurring viral, bacterial, or/and parasitic outbreaks.

*Piscirickettsia salmonis* is the etiological agent of Salmonid Rickettsial Septicemia (SRS), otherwise known as Piscirickettsiosis^34,35^. This organism is a Gram-negative facultative intracellular bacterium that is aerobic, pleomorphic, and non-encapsulated. This bacterium produces a systemic infection characterized by colonization of the kidney, liver, spleen, intestine, brain, and gills. SRS is one of the most severe infectious diseases plaguing the salmon industry owing to its highly contagious and aggressive nature^36^. There are often recurrent outbreaks and widespread transmission among and between farmed populations of cultivated salmon that are separated by great distances^37,38^. The virulence factors and pathogenic mechanisms of this genus remain poorly understood^39,40^. Whether there are members in other bacterial families cooperating with *Piscirickettsia* in the context of clinical SRS is not known.

The development of next-generation (NGS) DNA sequencing and bioinformatic tools has provided means to unbiasedly detect members of bacterial communities in distinct anatomical compartments of plants, animals and humans. Analyzing bacterial community structures affiliated with disease has been challenging because bacteria have traditionally been discovered or detected using a mixture of cultivation, histopathology, immunoreactivity or real-time polymerase chain reaction (RT-PCR) techniques. Many organisms will not be detected under these conditions because there is no prior knowledge, they cannot be grown in the lab or no assay exists. An alternative tactic uses oligonucleotides to PCR-amplify portions of the 16S ribosomal RNA (rRNA) gene locus conserved among bacterial phyla. This genomic locus contains segments of conserved and unique sequences that correspond to each different phylogenetic lineage. Amplifying multiple segments within the larger 16S rRNA genomic locus can increase sensitivity of detection. Sequencing these PCR products can collectively detect the genomic templates of most, if not all members of bacterial families present in complex mixtures. The quantity of DNA amplicons corresponding to each specific template in the sequencing data approximates the relative abundance of each bacterial member present in the original sample. These so-called ‘metagenomic’ data can be used to reconstruct the bacterial networks that were present in diseased and healthy samples. These reconstructions are generally limited to the taxonomic resolution of family. The 16S rRNA gene sequences registered in the data set represent only bacterial genomic templates. The bacterial templates detected using these NGS tools represent only part of a larger complex community that includes other marine microorganisms, algae, fungi, protists and viruses that collectively compose the complete microbiome located in specific anatomical compartments of the fish. Therefore caution must be exercised to not over interpret the data as representing all microorganisms potentially present in the sample. Despite these limitations, the quantities of all constituent bacterial DNA templates in the sequencing data can be ranked to identify taxonomic families that co-occur with statistical significance. These measurements define the detectable bacterial networks presumed to be present when the samples were collected. Routine surveillance of Atlantic salmon at the farming sites enabled us to collect samples that were well-matched. This circumstance was a unique opportunity to test if other taxonomic families co-occurred with *Piscirickettsia* in the gut and mucosa of SRS fish using these metagenomic techniques.

Orthogonal evidence for associations between members of different taxonomic families may be derived from the enrichment of metabolic pathways encoded by members of the bacterial networks. The metabolic enzyme content for each phylogenetic lineage may be retrieved from various databases (e.g., National Center for Biotechnology Information) to predict the composite metabolic activities potentially present in the microbial networks detected in the tissues. These metabolic pathways can be compared to predict biochemical activities that might be enriched or suppressed, for example in SRS-diseased relative to healthy Atlantic salmon. These reconstructions may help identify changes in metabolic profiles consistent with the changes observed in bacterial networks associated with infection. These bioinformatic approaches are not without the complications of false-positive results and sterility must be maintained at all points to prevent environmental contamination of the samples. However, this approach is a complimentary analysis that may help incriminate hitherto unknown associations between *Piscirickettsia* and other phylogenetic orders within the bacterial networks detected in sick fish.

Our goals were to measure the local bacterial communities of mid-gut digesta and lumenal mucosa of Atlantic salmon with and without clinical skin signs of SRS. We wanted to test if there was a shift in the composition of bacterial networks or predicted metabolic activities in SRS-positive versus healthy Atlantic salmon. The accuracy of these tools is sensitive to external factors that might imply false-positive predictions. We controlled external influences by accessing fish from farms cultivating Atlantic salmon of the same stock source, age, health-status, food provided and environmental habitat/temperatures. Having access to commercial Atlantic salmon collected during routine SRS surveillance created a unique opportunity for us to analyze fish from sites with equivalent farming and environmental conditions. We sequenced the bacterial 16S rRNA gene between the V4–V5 segments to survey bacterial constituents present in collected samples. We used phylogenetic analysis to explore whether there were differences in the bacterial network structure of fish with clinical SRS compared to healthy Atlantic salmon. At the same time, we analyzed whether members from other bacterial orders, or families, co-occurred with *Piscirickettsia* during clinical infection in the field. We reconstructed metabolic phenotypes based on unique bacterial profiles measured in the individual fish groups and searched for pathways enriched in SRS disease. We provide this survey as a resource for comparing other metagenomic investigations regarding bacterial fish pathogens. This knowledge could be used to develop new diagnostics, create therapeutic interventions, and ultimately improve fish health / farm management.

## Results

### Bacterial diversity and structure composition

**Figure 1:**
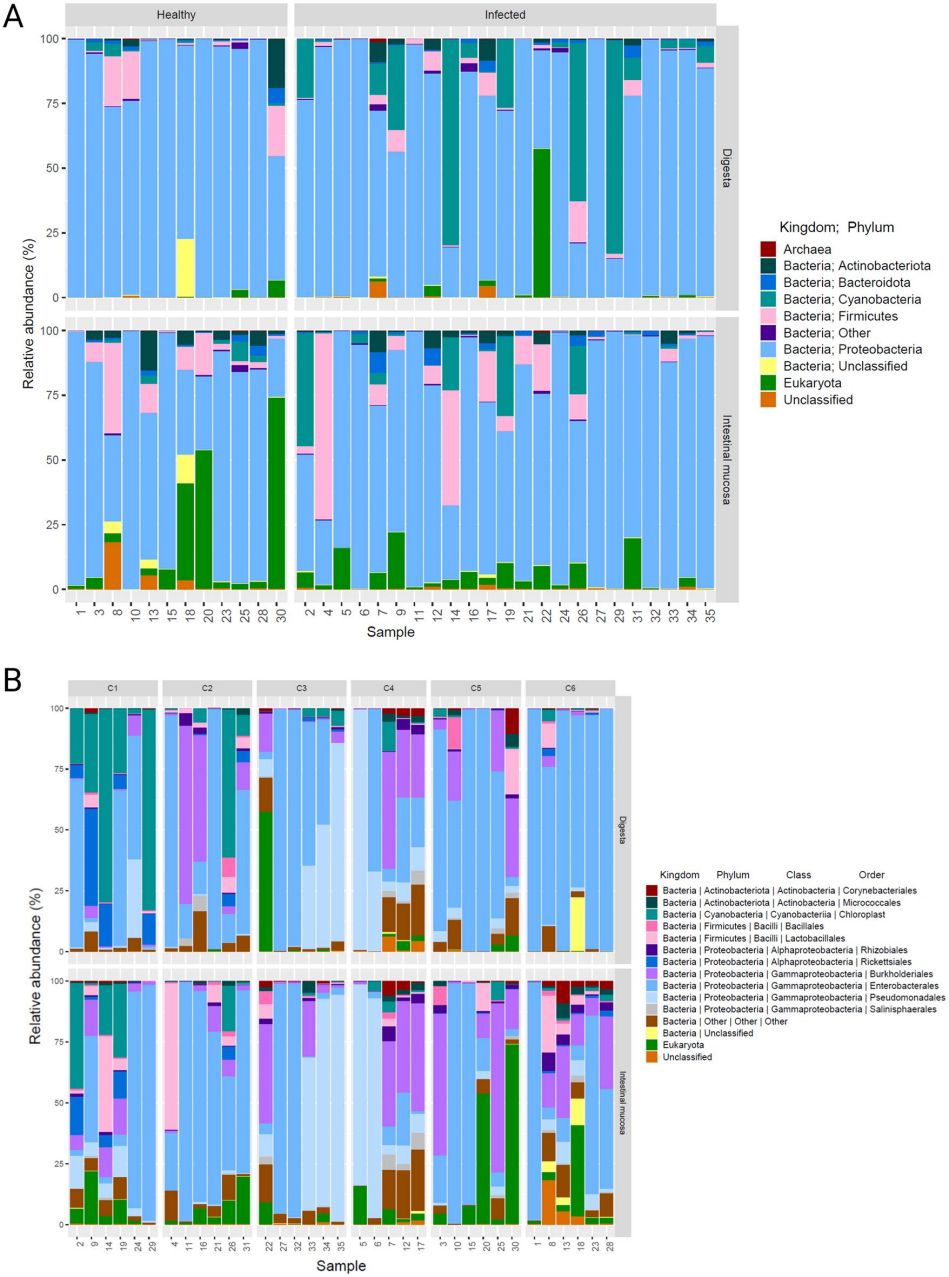
Illustration of the relative abundance of taxonomic profiles. **(A)** Relative proportion of phyla detected in healthy and SRS-positive salmon according the tissue sampled. **(B)** Relative abundance of taxonomic orders present in healthy and SRS-positive Atlantic salmon according to specific farm sites. Healthy fish were selected from sites C5 and C6 correspond to order shown in **(A)** while SRS positive fish were selected from farm sites C1, C2, C3 and C4 with corresponding order found in **(A)**. The contents of the digesta are shown on top and the contents of the mucosa are shown on the bottom. The phylogeny are color coded to indicate individual orders.

Skin and organ lesions immuno-histologically positive for *Piscirickettsia salmonis* is diagnostic for clinical SRS in Atlantic salmon. This diagnosis is typically confirmed by molecular detection of *P. salmonis* DNA by real-time PCR. We collected fish samples during naturally occurring outbreaks of SRS at four farm sites during 2021 (Supplementary Table 1). In each instance, the presence of *P. salmonis* in skin lesions was confirmed by histological and molecular diagnostic assays validated by the Chilean SENPESCA (Supplementary Fig. 1). We collected healthy fish from two separate farm sites known to be SRS-free and confirmed the absence of detectable *P. salmonis* on the skin of these samples using the same histological and molecular diagnostic assays. We focused on both the digestive material and mid-gut intestinal mucosa to assess internal bacterial communities because differences in microbiome composition between these two sites are believed to reflect colonization of cell surfaces and might have physiological consequences^2,41–43^. We extracted digesta and gut mucosa under surgically sterile conditions to minimize introduction of environmental microorganisms to the measured data. We PCR-amplified conserved DNA elements corresponding to the bacterial 16S rRNA gene locus spanning the V4–V5 regions. The nucleotide sequences located in this genomic region can be used to positively assign identity to > 90% of all known bacterial families^44^. We created dual-indexed DNA fragment libraries for each sample and mixed equimolar quantities to form a single pool. We sequenced the entire mixture on the Illumina platform and generated a repertoire of 35,547,801 raw sequencing reads. We removed low-quality reads and chimeras to yield 28,269,548 total reads and used this clean data to generate a total 7419 ‘so-called’ amplicon sequencing variants (ASVs) representing 34 phyla, 69 classes, 166 orders, 265 families, 602 genera, and 520 identified species (Fig. 1). We included an Excel table of the measured ASVs as Supplementary Table 1. The read counts of 16S rRNA gene amplicons were uniform across sample sets (Supplementary Fig. 2) and the rarefaction analysis indicated that the libraries were sequenced to a depth sufficient to capture the lowest abundant 16S rRNA gene amplicons (Supplementary Fig. 3). We verified there were no significant differences in the quantity of reads used to construct our ASVs that might have influenced our statistical power and confirmed congruence between ASV groups by comparing the computed Shannon diversity index of each sample set (Supplementary Fig. 4).

We computed the local diversity (α-diversity) of sequenced reads corresponding to the number of observed taxa and expressed these values as relative abundance of each phyla out of 100%^44^ (Fig. 1). We found that the *Proteobacteria* (synonym *Pseudomonadota*) (76.4 ± 25.1%) was the most abundant phylum in all the samples (Fig. 1A). *Piscirickettsia salmonis* is classified within this phylum. We observed *Firmicutes* as the second most dominant phyla in healthy (7.26 ± 10.13%) and infected (5.59 ± 11.61%) intestinal mucosa, but found that *Cyanobacteria* (15.24 ± 25.43%) was the second most prevalent phylum in the digesta of infected fish in contrast to *Firmicutes* (5.12 ± 8.40%) in healthy fish. Examination of the ASVs marked as *Cyanobacteria* indicated the DNA templates were likely derived from chloroplast origins. There were no toxic blooms of microalgae or *Cyanobacteria* recorded by the Chilean government at that time frame in the areas of the farm sites from which the fish samples were collected. There were quantitative differences in the bacterial orders detected in the lumen mucosa compared to gut digesta in the healthy fish 2, 4, 11, and 19 (Fig. 1B). This observation hints that the structure of resident bacterial networks in the mucosa may be isolated from the digesta. Positive growth associations or cooperative production of biofilm may be a mechanism that isolates the lumen surface from the luminal contents, and would account for the lack of *Cyanobacteria* ASVs in the mucosa of the same fish (Fig. 1B). There was broad agreement in the abundance of microbial orders detected in the healthy and SRS-positive fish (Supplementary Fig. 5). We did not calculate a significant difference between the ASVs representing taxonomic orders between the healthy and sick fish groups (Supplementary Fig. 5) when using the Mann–Whitney test.

### Differences in abundance of bacterial constituents

**Figure 2:**
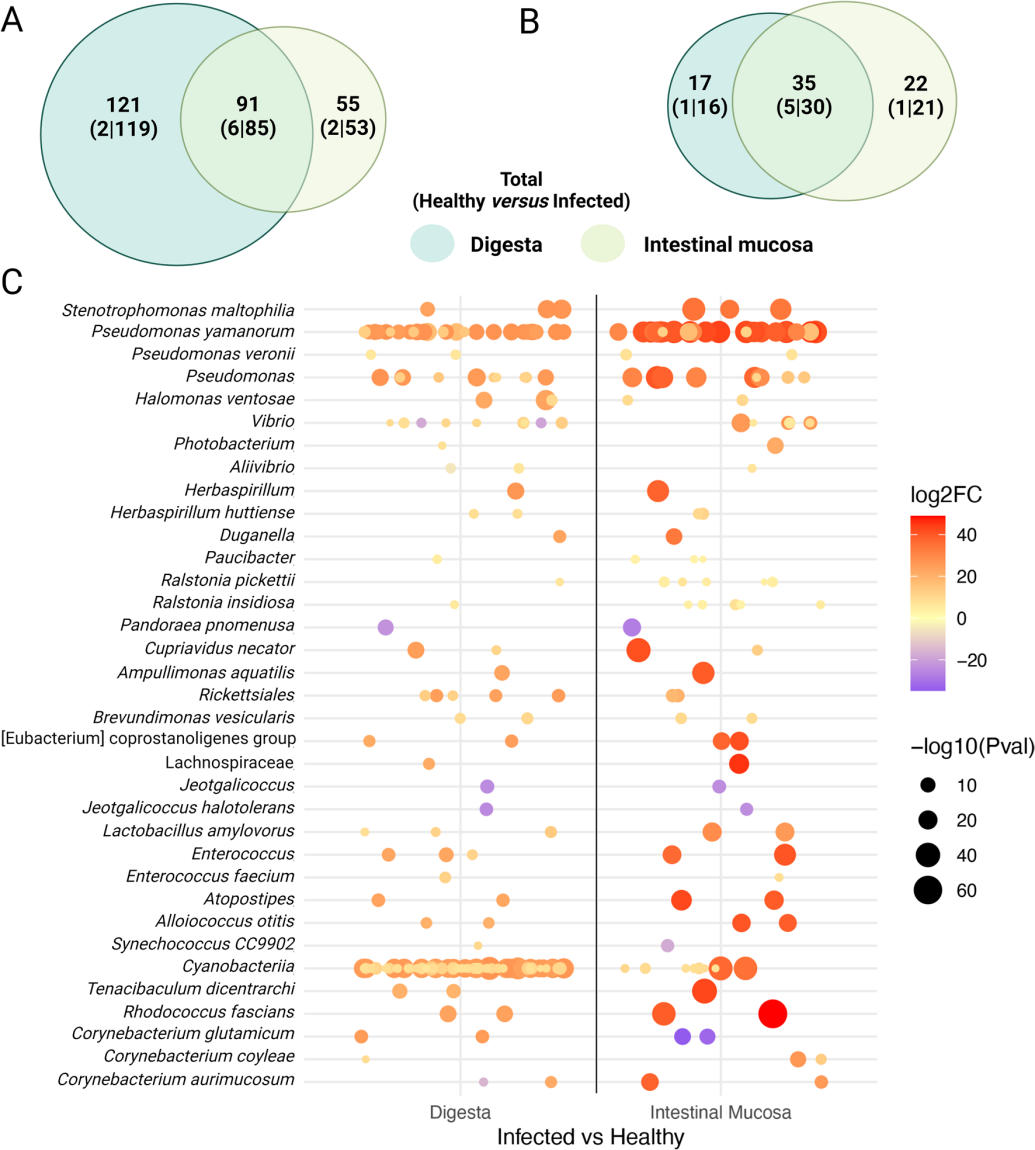
Differential abundance of amplicon sequencing variants (ASVs) in the digesta and intestinal mucosa of healthy and SRS-positive Atlantic salmon. **(A)** Venn diagram of differentially abundant ASVs in digesta and intestinal mucosa of healthy and SRS samples. The top number indicates the total number of ASVs in that group. The number of ASVs derived from healthy and SRS positive fish are shown in parentheses. Contents of the digesta and mucosa are shown in green and yellow, respectively. **(B)** Venn diagram of differentially abundant ASVs in which we obtained a taxonomic assignment. The number of ASVs derived from healthy and SRS positive fish are shown in parentheses. Contents of the digesta and mucosa are shown in green and yellow, respectively. **(C)** The assigned taxa of differentially abundant ASVs comprising the 35 genera detected in **(B)** above. Each circle is a particular Amplicon Sequence Variant (ASV) identified and assigned to a particular species. There can be more than one ASV for a particular species, because each ASV represents a cluster of a group of reads representing a single DNA sequence recovered from the sequencing data. In this figure, each of the dots represents one of these ASVs within the particular taxonomic assignment. Fold-change (log_2_FC) is represented in the logarithmic scale of base 2, red color means overabundance in infected samples, and blue represents overabundance in healthy samples. Adjusted p-values are represented in the negative logarithmic scale of base 10. Bigger circles mean higher p-value statistical significance.

To investigate if there were any quantitative differences in the bacterial networks detected in SRS fish, we used the Divisive Amplicon Denoising Algorithm 2 (DADA2) R-package to evaluate the abundance of ASVs present in each sample and calculate differences in the amounts of phyla comprising each bacterial community (Fig. 2). We found 212 ASVs in the digesta and 146 in the gut mucosa that were differentially abundant when comparing infected and healthy specimens (Fig. 2A). We found 17 ASVs overrepresented in the infected digesta and 121 overrepresented in the healthy digesta samples. In the case of the intestinal mucosa, we found 22 ASVs in the infected and 55 in the healthy samples. In total, we found 91 ASVs as differentially abundant in both of the compared conditions, with 121 (2 healthy and 119 infected) exclusive for the digesta and 55 (2 healthy and 53 infected) for the intestinal mucosa. From these sets of differentially abundant ASVs, we were able to retrieve taxonomic assignment of 74 taxa, with 17 of them specifically in the digesta, 22 in the intestinal mucosa, and 35 shared within both comparisons (Fig. 2B). We found that quantities of species in the genus *Pandoraea* were depleted in the infected mucosa, while species in the genus *Jeotgalicoccus* were depleted in healthy mucosa. *Corynebacterium aurimucosum* was enriched in SRS-positive mucosa while *Corynebacterium coyleae* and *Corynebacterium glutamicum* were enriched in the mucosa of healthy Atlantic salmon (Fig. 2C). We list the complete set of differentially abundant ASVs corresponding to bacterial families that co-occurred with *Piscirickettsiaceae* (with associated p-values < 0.05) in Supplementary Table 2.

To test whether *P*. *salmonis* was present in the guts of the sampled fish, we surveyed the data sets for the presence of ASVs corresponding to this species. We expected to find *P*. *salmonis* 16S rRNA gene sequences in the guts of SRS-positive fish but we surprisingly found *P. salmonis* ASVs in the digesta and gut mucosa of three healthy control fish (Supplementary Fig. 6). *Piscirickettsia salmonis* was detected in the gut mucosa in almost all the SRS-positive fish collected from sites C3 and C4, and in at least one fish from farm sites C1 and C2 (Supplementary Table 1). This measurement showed that half of the fish positive for invasive *P. salmonis* in their skin did not have detectable *P. salmonis* in their mid-gut.

To evaluate if other bacterial pathogens were present in gut communities of these SRS-positive fish, we surveyed the data for ASVs corresponding to species in the *Tenacibaculum*, *Aeromonas*, and *Renibacterium* genera (Supplementary Fig. 6). To our surprise, we detected *Aeromonas salmonicida* and *Renibacterium salmoninarum* in five of the twelve SRS-free control fish. We did not detect *Tenacibaculum maritimum* in any control fish. All of these species were detectable in more than half of the SRS-positive fish, but again, some of the fish presenting with clinical SRS on their skin did not have any detectable *P*. *salmonis* or other bacterial pathogen in their mid-guts.

### Taxa associated with *Piscirickettsiaceae*

The concept that members of a localized bacterial network cooperate to promote pathologic outcomes predicts there would be stimulatory and inhibitory interactions between so-called ‘driver’ taxa and other organisms in the network. The bacterial community in the gut of healthy Atlantic salmon would have constituent members that would quantitatively increase or decrease as the bacterial network shifted toward a state of disease. Other species may opportunistically grow under these conditions to become major constituents of the community population in the diseased state. The increase or decrease of individual members in the network is indicative of organisms that ‘shift’ the community structure from one state to the next. These so-called ‘driver’ species are predicted to have higher or lower co-occurrence rates with a species of interest; in the current case, *P. salmonis*.

**Figure 3:**
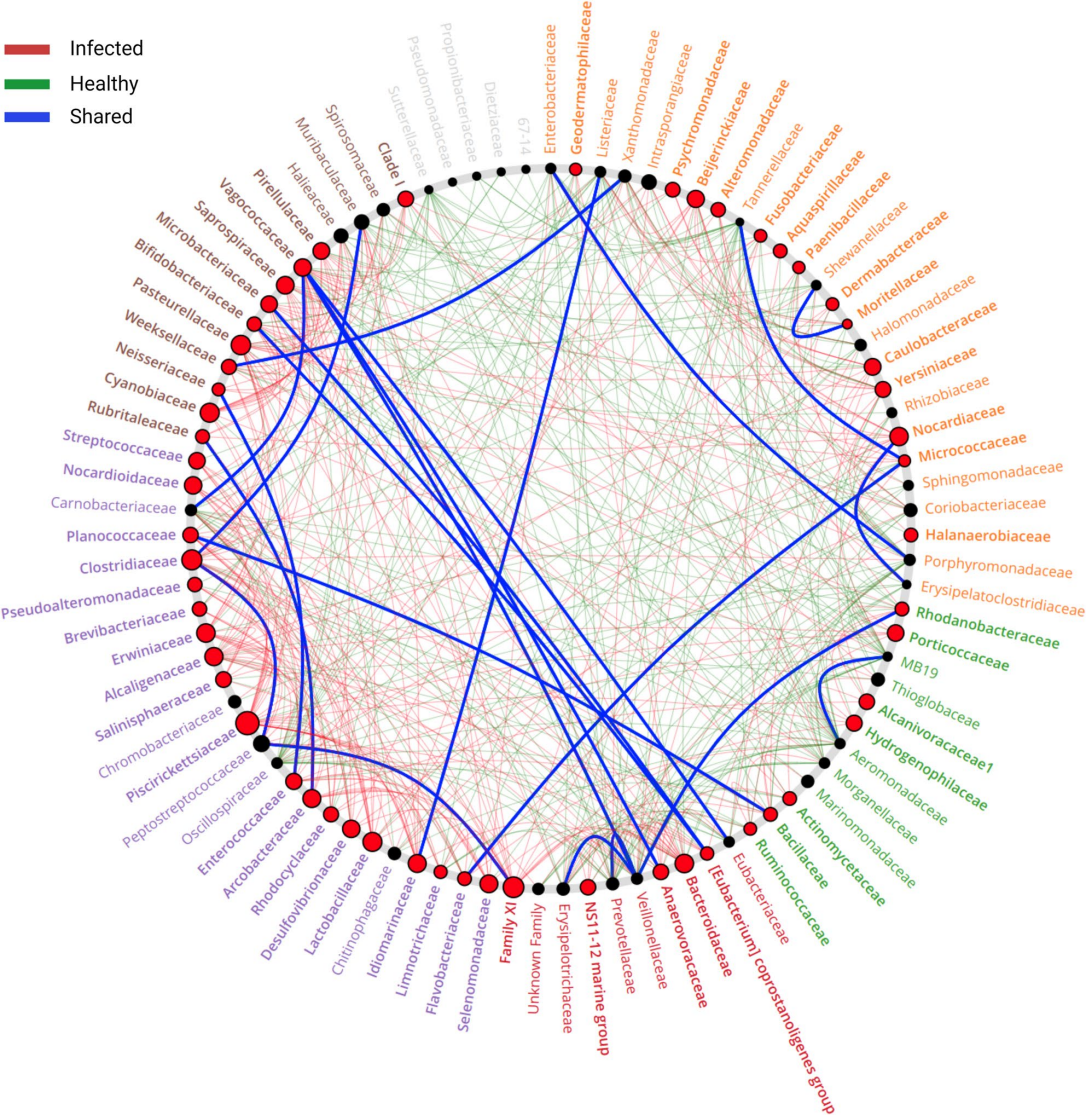
Community shuffling plot of the changes in bacterial network structures of SRS-positive compared to healthy Atlantic salmon. This plot illustrates the relationships between members of the bacterial families found in the fish. This graphic consists of nodes arranged in a circle. Each node represents a member of the bacterial network. The co-occurrence between bacterial network members of the infected fish are indicated by a line connecting the two specific nodes. This line is called an ‘edge’ and red, green and blue indicate associations enriched in the SRS-positive, healthy and shared bacterial networks, respectively. This graphical output highlights the sub-networks and ‘driver’ nodes. All nodes belonging to the same community are randomly assigned a similar color. Grayed-out nodes represent the ones present in both but directly interacting with the common sub-network in either infected or healthy conditions. Node sizes are proportional to their scaled NESH score, as calculated using NetShift tool. A node is colored red if its proximity increases from healthy to infected samples. Nodes that are big and red indicate that these organisms are significant ‘drivers’ in the shift from healthy to a SRS-diseased state in the localized bacterial communities.

To explore the possibility that other bacteria might associate with *Piscirickettsiaceae* to promote SRS in Atlantic salmon, we measured the pair-wise associations between each ASV in healthy and SRS fish using the NetShift web tool to construct ‘co-occurrence’ networks of bacteria detected in our samples^45^. This application calculates a ‘Neighbor Shift Score’, or NESH value, for pair-wise interactions between each ASV of the ‘case’ and ‘control’ groups. In our present study, the SRS and healthy fish were the case and control groups, respectively. This NESH score estimates the degree of co-occurrence between any two species in the network, which may be positive, negative or neutral, however our intent was to detect interacting members that are enriched in SRS compared to healthy fish. We constructed networks for both the healthy and diseased fish and found significant shifts in the bacterial network structures of the diseased fish compared to healthy. We graphically represented this information in the form of a ‘Network Shift Plot’ to illustrate positive or negative associations between members of the bacterial communities in the same graphic (Fig. 3). We found that the bacterial communities of the SRS-positive fish underwent large-scale changes in the abundances and organisms present compared to healthy fish. These changes included distinct co-occurrence networks with unique properties regarding the number of bacterial species (91 healthy and 86 infected) as well as the number of associations detected between members in the networks (305 and 247 for healthy and sick, respectively). The number of edges that are exclusive to either network is an indicator of extensive reorganization within the community. Interestingly, the bacterial networks contained a high number of associations between species that were exclusive to each condition, with 224 present only in healthy samples and the other 282 in diseased specimens. This observation is consistent with a significant reconfiguration in the interactions established by the bacteria in the SRS-positive compared to healthy Atlantic salmon.

To further explore the associations of *P. salmonis* with other organisms in the common ‘infected’ sub-network, we evaluated the positive and negative associations between the *Piscirickettsiaceae* family and other families using the co-occur tool embedded in the NetShift algorithm. This tool calculates the statistical significance of the Spearman correlation between any two taxa present in the network by re-sampling null distributions of correlation values (1000 iterations). This tool illustrates the co-occurrence network as a series of nodes connected by lines and is used to illustrate the overall organization of the bacterial community and enables visual assessment of its modularity in Atlantic salmon with SRS. An association between two taxa is indicated with a line connecting the two nodes, which is called a path. The average path length is the number of steps necessary to travel from one node to another in the network and reflects how compact the bacterial network is. The density of a node indicates the proportion of bacterial associations (edges) out of all theoretically possible associations. A greater node density indicates an increased level of communication between bacterial members present in the network.

**Figure 4:**
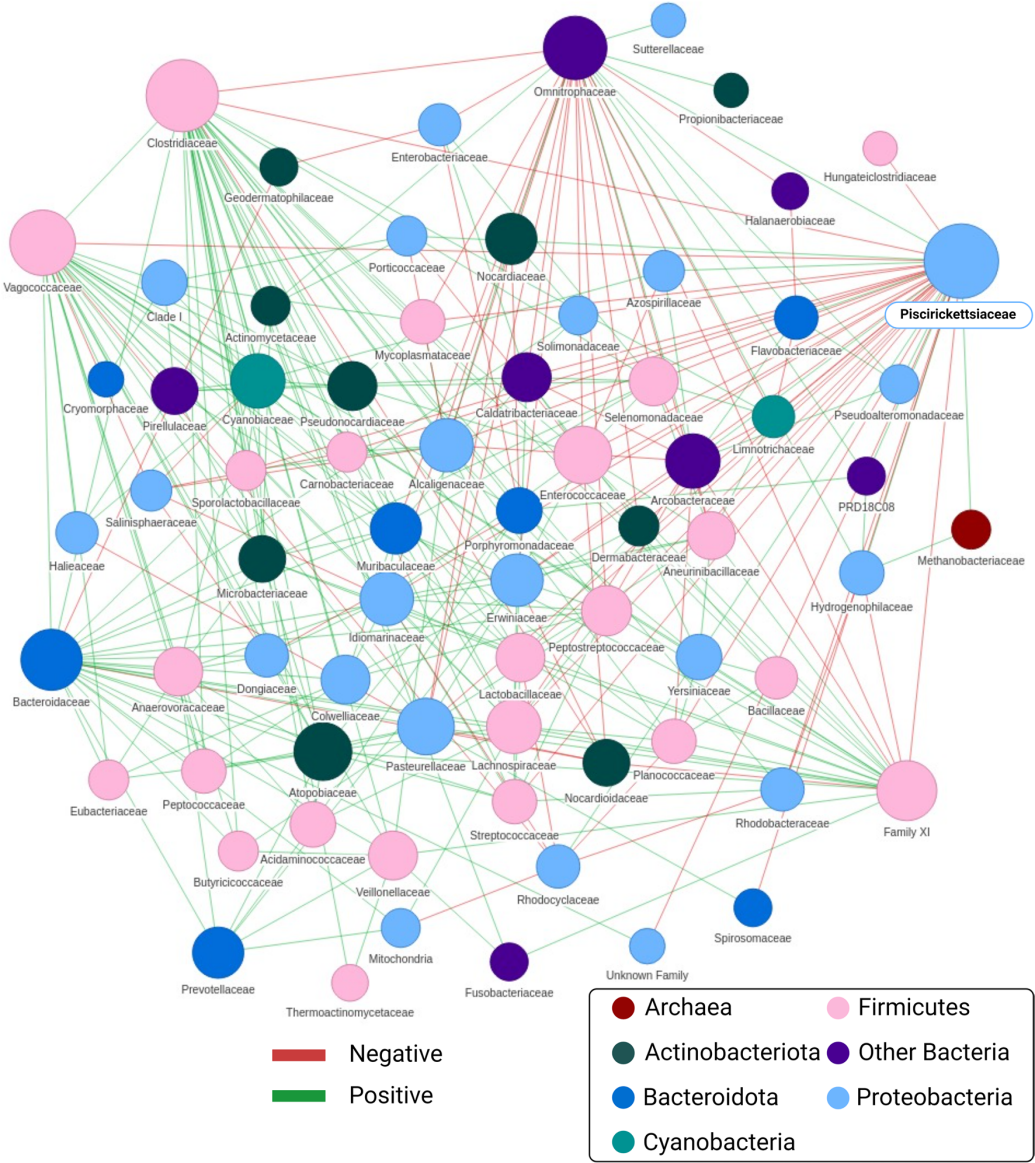
Illustration of co-occurrence network in SRS-positive salmon. This plot illustrates the statistically-significant (p ≤ 0. 005) associations detected between *Piscirickettsiaceae* and other microbial families. Line color indicates the type of interaction with red lines representing negative associations (occurring less than expected), and green lines representing positive associations (occurring more than expected). The density of a node relates to the number of other members the organism communicates with. The length of the line represents the closeness of the association between bacterial members. We highlighted the *Piscirickettsiaceae* for easy identification.

We calculated an average path length of 3.07 and 2.65 and a density of 0.083 and 0.060 for healthy and infected Atlantic salmon, respectively. (Fig. 4). This observation indicates that there was closer connectivity between the bacteria in the SRS-positive fish even though there are fewer associations between bacterial driver species. This data is consistent with the interpretation that the bacterial communities are less diverse but transfer higher amounts of information between members of the network in the SRS-positive fish, which might indicate colonization events. This analysis identified a list of 27 families having negative associations with *Piscirickettsiaceae* in infected fish and another set of 10 families with positive associations, i.e., associations consistent with the transition from a health-associated bacterial community to an SRS-associated community (Supplementary Table 2). Interestingly, one of the negative associations is with members of the family *Lactobacillaceae*, which makes sense given the reported probiotic properties of this family.

The dissimilarities between bacterial communities can be compared to provide information about the relatedness between different ‘physiological states’ of the host. Instead of looking at relationships between *Piscirickettsiaceae* and other microbial constituents present in the bacterial networks of healthy and sick fish, we explored how the gut ASVs of healthy fish compared to the ASVs of SRS-positive fish. We calculated the nonmetric multidimensional scaling (NMDS) of the ASV dissimilarities in all samples of the 16S rRNA gene amplicons according to the Bray–Curtis method (Supplementary Fig. 7). This tool graphically illustrates the dissimilarities between bacterial networks in a two-dimensional space. We calculated that the bacterial networks of digesta and gut mucosa in healthy fish are, overall quite similar. Consistent with our findings shown in Supplementary Fig. 6, the complexity of the bacterial networks in some of the SRS-positive fish overlapped with that of the healthy fish, indicating the infection in the skin did not necessarily disrupt bacterial networks in the gut. The bacterial networks found in the gut of SRS-positive fish exhibited dissimilarity with the composition of healthy fish, which is broadly consistent with a general state of dysbiosis. We performed a Permutational analysis of variance (PERMANOVA) to test if the bacterial community structures (ASV profiles) for the four subject groups (healthy digesta, infected digesta, healthy intestinal mucosa and infected intestinal mucosa) were significantly different. We used the adonis2 function (Permutational Multivariate Analysis of Variance Using Distance Matrices) from the vegan R package to calculate an F value for the four groups of 2.0663, and a p-value of 0.002 (Supplementary Information Excel file). This finding indicates that the variation between groups was higher than the variation within groups. However, this analysis reaffirms the interpretation that healthy Atlantic salmon have a particular gut bacterial network and that many of the SRS-positive fish deviate from this network structure.

### Metabolic phenotypes in *P. salmonis* infection

**Figure 5:**
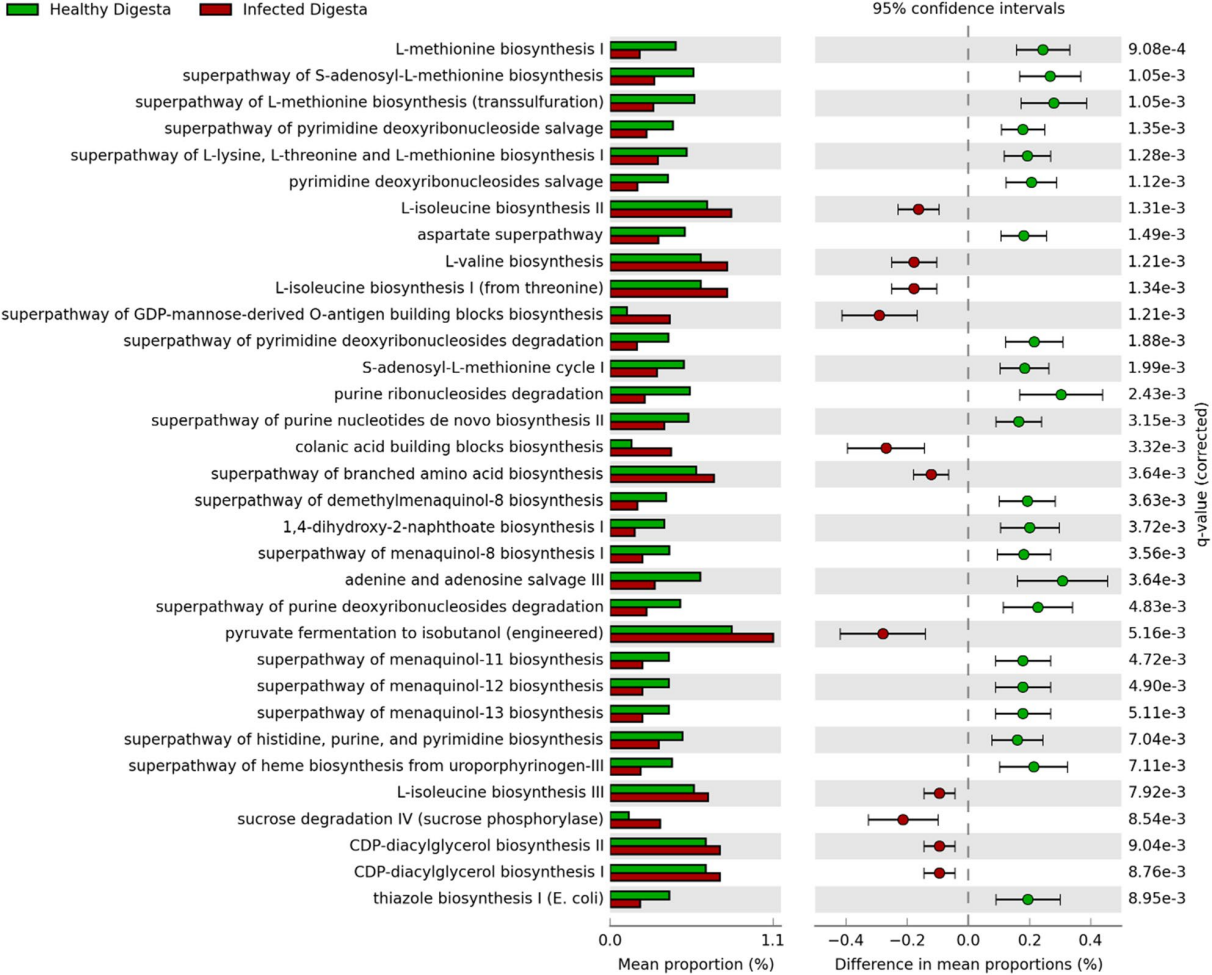
Metabolic phenotypes predicted to exist in health and SRS-positive salmon based on unique bacterial network compositions. Shown on the left are the metabolic pathways detected with statistical significance (q-value). The mean proportion of organisms expressing specific metabolic pathways in the digesta of healthy and sick fish are shown as horizontal bar graphs in green and red, respectively. The percentage differences in mean proportion of each metabolic phenotype is shown as a horizontal spread for healthy and sick salmon, where the error bars indicate the 95th percentile. This analysis was performed using the PICRUSt2 tool.

Metagenomics data represents an inventory of the microorganisms present in a sample^17^. The PICRUSt2 bioinformatics pipeline can recover the genome sequences of known species linked to the specific 16S rRNA gene elements present in the sequencing data^46^. This program extracts the metabolic pathways encoded by these recovered genomes to compute a ‘metabolic phenotype’ based on the constituent members of a given bacterial community. We used this tool to calculate whether any metabolic pathways were enriched in clinical SRS fish and found significant differences to those pathways enriched in healthy fish. In total, 414 metabolic pathways were predicted, and 33 pathways were calculated as significantly different between healthy and clinical SRS (Fig. 5). The three most enriched pathways in infected digesta were the “super-pathway of GDP-mannose-derived *O*-antigen building blocks biosynthesis”, “colanic acid building blocks biosynthesis”, and “pyruvate fermentation to isobutanol (engineered)”. These predictions posit the existence of discontinuous pathways in two or more organisms. We observed the “super pathway of L-methionine biosynthesis (transsulfuration)”, “purine ribonucleosides degradation,” and “adenine and adenosine salvage III” in healthy fish. Our analysis detected statistically significant differences in the pathways identified only in digesta-derived samples. This observation indicates that these pathways might be conserved in the mucosa and are unperturbed despite an external SRS infection.

## Discussion

There is a lack of knowledge about bacterial network-gut-fish host interactions but our work adds to a growing body of evidence describing these features in salmonids^47^ and other farmed marine species^48^. Preventing exposure to pathogenic species or vaccination is thought to be sufficient to protect fish health in the aquaculture farm environment. However, these assumptions are increasingly questioned as new evidence indicates that cooperation between members of resident bacterial networks may be equally important for host defense. We found *Aeromonas salmonicida*, *Renibacterium salmoninarum*, *Tenacibaculum maritimum* and *Piscirickettsia salmonis* in the gut of healthy Atlantic salmon. The simple interpretation of this observation is that exposure to these pathogens may be insufficient to allow colonization and disease. Our analysis of the metagenomics data indicated there may be interactions between bacterial members of two or more taxonomic families to create a metabolic phenotype that favors *P. salmonis* virulence. This evidence is not causal, but provides new directions to investigate virulence mechanisms at the bacterial network level that may broadly impact aquaculture practices.

We took extensive care to minimize sampling variations to ensure quality measurements of bacterial networks. Fish samples are often collected and transported great distances in which variation in stabilization, temperature and handling can promote contamination or degradation of nucleic acids. This variation creates real uncertainties as to whether the content represented the original bacterial network. We ensured the farming and environmental parameters at the collection sites were as equal as possible to minimize any influence on the physiology of the fish. We took a mobile surgical unit to the farm sites and collected samples and immediately stabilized the samples for targeted V4–V5 sequencing. The quality statistics of our read content and similarities between DNA fragment libraries support the interpretation that individual DNA fragment libraries were quantitatively equivalent (Supplementary Figs. 2, 3).

The phylum *Proteobacteria* was a dominant member of the bacterial community. This finding was not surprising given that this phylum is reported to be the most abundant in many marine and freshwater fishes^49,50^, including Atlantic salmon (*S. salar*)^18,50^. Taxa belonging to *Proteobacteria* express metabolic pathways catalyzing carbon and nitrogen fixation, and stress response signaling^46^. They are crucial to the overall digestive process in fish^51^ and the abundance of organisms in the phyla *Actinobacteria*, *Firmicutes*, and *Proteobacteria* in our samples were consistent with previously reported intestinal bacterial networks of Atlantic salmon^52,53^. Pelusio et al. measured similar values in other salmonid species that included Rainbow trout (*Oncorhynchus mykiss*), in which the most abundant taxa were also from the *Actinobacteria*, *Firmicutes*, and *Proteobacteria*, albeit in different ratios than our measurements^54^. These three phyla represented about 98% of the total intestinal bacteria previously reported, and were essentially consistent with many of the fish we observed. The phylum Bacteroidota was represented by species in the pathogenic genus Tenacibaculum (Supplementary Fig. 6).

We found that the abundance of *Firmicutes* was lower than the proportion of *Proteobacteria* in sick fish than in healthy. We point out that the *Firmicutes* phylum is reported to be among the most common and metabolically important in the gastrointestinal tract of several fish species^55,56^. We found that the orders *Bacillales* and *Lactobacillales* within the phylum *Firmicutes* were present, and the latter were within the lactic acid bacteria (LAB) group. The LAB were previously identified in the gut microbiota of Atlantic salmon where they are presumed to have beneficial effects on the host’s immune regulation, digestive processes, and the inhibition of certain pathogens under specific conditions^57,58^.

We were surprised to detect ASVs corresponding to *P. salmonis* in the healthy control fish, which included digesta and mucosa. Equally curious was the detection of ASVs corresponding to other pathogenic species in these controls. However, the abundance of these pathogen ASVs were trace amounts relative to the total quantity of ASVs, and second, they were only detected in three out of 24 fish. These pathogens are widely distributed in the environment^59^. Our observations are not wholly unexpected because *P. salmonis* is endemic to the coastal waters surrounding Chile and there are frequent outbreaks of SRS throughout the year. At least four explanations account for our observations. First, the fish might be constantly exposed to *P. salmonis* but at concentrations lower than the minimum dose necessary to cause infection. Second, commensal bacteria, such as lactobacilli, may competitively inhibit *P. salmonis* from colonizing gut mucosa and thereby protect salmon from infection. This explanation is widely supported by the literature, and interestingly, the high microbial diversity observed in the mucosa of healthy fish is consistent with that reported by other investigators. Third, the presence of *P. salmonis* in the gut might indicate a ‘healthy’ carrier state that has until now been hitherto unrecognized. It is noted that SRS-free salmon exhibited no skin lesions and tested clinically-negative for *P. salmoni*s in the skin (Supplementary Fig. 1). Fourth, perhaps the three healthy fish positive for *P. salmonis* were at the earliest stages of infection, and in two weeks would appear similar to the sick fish. Longitudinal studies of SRS infection in relation to gut bacterial communities could test if these explanations are valid.

The presence of other known bacterial pathogens in the healthy fish is equally intriguing. The detection of ASVs corresponding to pathogens other than *P. salmonis* in the healthy controls demonstrates the sensitivity of NGS methods. This finding highlights a hitherto unappreciated prevalence of these pathogenic bacteria in healthy salmon. This observation reinforces the notion that mucosal barriers on gut luminal surfaces play a critical role in host defense, and it seems that resident bacterial networks likely contribute to the integrity of mucosal compartments. We do not believe *P. salmonis* ASVs, or other pathogen ASVs, detected in our healthy fish were the result of environmental or laboratory contamination. Great care was taken to process these samples in a clean and sterile laminar flow hood. Separate protocols were performed in dedicated laboratories. Quality controls showed these tactics minimized the possibility of contamination during genomic 16S rRNA gene amplification, above.

Karlsen et al. analyzed another ulceration outbreak and its links with the gut bacterial network at a *S. salar* farm in the north of Norway^60^. They reported that the detectable bacterial networks in different fish were broadly consistent, primarily including members of the *Mycoplasmataceae* (67.9–98.6%) family. These authors also detected *P. salmonis* and *Francisella noatunensis* subsp. *Noatunensis* in healthy fish*,* which constituted 0.18% and 0.005% in the fish samples, respectively. These findings are consistent with our observations, and indicated that *P. salmonis* may be present at low, trace amounts without causing disease. Our detection of *Tenacibaculum*, *Aeromonas* and *Renibacterium* in ostensibly healthy control fish supports the idea these organisms did not exceed the minimum infectious dose necessary to cause infection.

Species within the *Piscirickettsiaceae* family exhibited both positive and negative co-occurrence relationships with members of other bacterial families in SRS-positive and healthy fish. Nine taxonomic families registered positive interactions with *Piscirickettsiaceae*. It was interesting to observe that the *Flavobacteriaceae* family*,* in which several *Tenacibaculum* species are found^60,61^, was well represented in the ASV data. Amplicon sequence variants corresponding to the species *Tenacibaculum dicentrarchi* were detected in the digesta and intestinal mucosa of salmon with clinical SRS (Supplementary Fig. 6). This finding is interesting because this species is thought to be primarily a skin pathogen. However, our data suggests that this organism, and perhaps others believed to be external to the fish gut, might effect changes on internal mucosal membranes.

We predicted that many metabolic pathways were enriched in healthy fish. These examples included amino acid biosynthesis and nucleotide salvage/degradation. Interestingly, we found two ‘engineered’ or otherwise ‘chimeric’ metabolic pathways to be enriched in the SRS-positive fish. This designation indicated that the complete pathway does not naturally occur in any single known organism (e.g., the super-pathway of GDP-mannose-derived O-antigen or the pyruvate fermentation to isobutanol pathway, respectively) and is possibly the result of growth cooperativity between members of two or more bacterial taxa. Another interesting pathway enriched in the SRS-positive fish related to virulence was colanic acid. This pathway is associated with adhesion of pathogenic bacteria to cell surfaces^62,63^. The overall conservation of metabolic phenotypes predicted in healthy and sick fish suggests that clinical SRS of the skin might not significantly perturb the gut mucosa. However, this may be premature because we do not know when each fish was infected. The level of colonization might depend on the progression of disease. Some of the sick fish may have been infected longer than others and the samples we collected may be from fish at different stages of disease.

The minimum infective dose (MID), or threshold of quorum, for *P. salmonis* to cause disease is not known but our data from healthy fish indicates that no greater than 0.01% of the total microbial content. Homoserine lactone derivatives are the major class of quorum sensing molecules in Gram-negative bacteria but whether *P. salmonis* uses these signaling molecules remains controversial^7,64^. The lactone ring is derived from S-adenosyl methionine and this pathway was enriched in healthy fish, which makes sense given that these phyla consist of Gram-negative organisms. One possibility is that exposure to environmental or bacterial-derived toxins might lower the MID needed for *P. salmonis* to initiate infection, although that conclusion is not possible to reach given our limited data.

This work reinforces the view that a comprehensive understanding of intestinal dysbiosis in relation to *P. salmonis* infection requires a metagenomics approach. We found evidence that *P. salmonis* and bacteria in other taxonomic families were enriched in fish with clinical disease and that healthy fish contained distinctly different bacterial networks at the same anatomical sites. Our collective findings are remarkable for several reasons: First, they support the idea that a comprehensive understanding of pathophysiology requires looking at bacterial communities, not just a single pathogen. Second, these data establish a baseline for the occurrence of four economically-important bacterial pathogens in the gut of healthy salmon. This information will be important for calibrating new diagnostics based on next-generation sequencing. Third, these data provide more evidence to support the conclusion that metabolic phenotypes might result from cooperativity between organisms in different taxonomic orders and suggests that perturbing cooperative interactions instead of individual pathogens might mitigate infectious outbreaks of *P. salmonis*. This work shows that detecting trace amounts of *P. salmonis* does not necessarily indicate the fish are clinically sick. However, many interesting questions remain unanswered, especially related to the time course and cooperativity between different taxonomic families. For example, it is not known if the healthy control fish in which pathogens were detected were at the earliest stages of disease. A longitudinal study investigating the time course of bacterial network changes in various anatomical sites might answer this question. Our findings help predict health outcomes based on the frequency of detecting specific genera and potentially establish cut-off thresholds for minimum quantities of bacteria necessary to cause disease. Greater numbers of fish samples would increase our statistical power, but overall, these preliminary evidence support the idea that gut dysbiosis is associated with clinical SRS in Atlantic salmon.

## Methods

### Ethics declaration

The Centro de Investigaciones Biológicas Aplicadas (CIBA, located in Puerto Montt, Chile) is authorized to perform diagnostic services for aquaculture purposes, and the associated experimental protocols were approved by the Chilean National Fish and Aquaculture Service (Servicio Nacional de Pesca y Acuicultura de Chile, http://www.sernapesca.cl/). All protocols complied with Chilean law (Ley 20.380 de Chile sobre Protección de Animales) concerning animal protection in the pursuit of biomedical investigations and are substantially based upon, and consistent with, the European Union Directive 2010/63/EU revising Directive 86/609/EEC on the protection of animals used for scientific purposes, which was adopted on September 22, 2010. We took advantage of regularly scheduled surveys in which farms were assayed for disease. None of the fish were collected solely for the purpose of this investigation and required no additional ethical review beyond the existing authorization regulated by the Servicio Nacional de Pesca y Acuicultura of Chile. Fish were sacrificed using anesthetic overdose (20% benzocaine) until onset of rigor mortis according to Annex IV of the EU Directive 2010/63/EU, above. We did not perform any procedure on live fish. The use of tissues collected herein were maximized to refine, reduce, and replace animal experimentation according to the sentiment this directive. Transportation of samples and compliance with statutes was overseen by the Servicio Nacional de Pesca y Acuicultura de Chile and Biomedical Research Ethics Committee of the Universidad San Sebastian. The authors confirm these procedures complied with ARRIVE guidelines (www. https://arriveguidelines.org/).

### Sampling of salmonids

We analyzed Atlantic salmon (*S. salar*) weighing 2869.34 g (± 3160.42 g) in the grown-out phase collected from four Chilean salmon farming sites located in the Los Lagos and Aysén Regions: C1, C2, C3, C4, C5 and C6. The sampling scheme is presented in the Supplementary Table 1. Briefly, we collected 23 digesta and 23 lm samples from fish that exhibited clinical SRS at sites C1, C2, C3 and C4 during outbreaks in the month of January 2022. We collected an additional 23 digesta and 23 lm samples from SRS fish at the same farm sites during continued outbreaks in the month of February 2022. In total, we collected 46 digesta and 46 lm samples from SRS positive fish. We collected a total of 24 digesta and 24 lm samples from healthy fish located at farm sites C5 and C6 during the months of January and February 2022. All SRS cases were characterized by ulcerative lesions at the cutaneous level with a focal to multifocal distribution without detectable ulcers in internal organs. We confirmed these fish to be positive for *Piscirickettsia salmonis* DNA using clinically-validated real-time polymerase chain reaction (PCR) assays following the methodology reported by Karatas et al.^65^ according to reporting standards of the Servicio Nacional de Pesca y Acuicultura de Chile. We analyzed twelve healthy fish from two separate farm sites (C5 and C6) that did not present clinical signs of SRS. We confirmed that these control Atlantic salmon (*S. salar*) were not infected with *P. salmonis* by applying (i) real-time PCR calibrated against authentic positive controls and (ii) histological analyses of the organs (kidney, liver, and spleen). Gram staining and microbiological cultures were performed according to standard clinical methods to confirm that the fish were negative for SRS infection. We aseptically collected intestinal digesta material and intestinal mucosa using surgically clean conditions. We practiced exacting aseptic technique in a laminar flow hood to ensure that each sample was collected under similar conditions. We used freshly isolated samples and equalized the time between sample collection and metagenome amplification to minimize any differences in time experience between collections from each fish.

### DNA isolation, 16S rRNA gene amplification, and sequencing

We extracted DNA from 1 mg of each isolated digesta and mucosal tissue sample using the Qiagen DNA Microbiome kit according to the manufacturer’s protocol. DNA concentration was measured using 2 µl of samples through the Invitrogen Qubit dsDNA BR Assay kit (Invitrogen Qubit 4 Fluorometer). Isolated DNA was used for amplicon sequencing, following the betas® DNA approach. Two hypervariable regions (V4 and V5) of the bacterial 16S rRNA gene were amplified using aliquots of the isolated DNA from each sample using the primer set: 515F (5ʹ-GTGYCAGCMGCCGCGGTAA-3ʹ)/806R (5ʹ-GGACTACNVGGGTWTCTAAT-3ʹ). Each sample was subjected to a 35-cycle PCR using the HotStarTaq Plus Master Mix Kit (Qiagen, Valencia, CA) under the following conditions: 95 °C for 5 min, followed by 35 cycles of 95 °C for 30 s, 53 °C for 40 s, and 72 °C for 1 min. After that step, a final elongation step was performed at 72 °C for 10 min. Next, all amplicon products from different samples were mixed in equal concentrations and purified using SPRI beads. Samples were sequenced using the Illumina NovaSeq platform by following the manufacturer’s protocols (Illumina DNA prep kit and Nextera Library XT DNA preparation kit). The datasets generated and analyzed during this investigation are available at the Sequence Read Archive (SRA) repository at the National Center for Biotechnology Information (NCBI) under the reference number PRJNA895462 and accessible at this link: https://www.ncbi.nlm.nih.gov/sra/PRJNA895462.

### Data filtering, production of amplicon sequence variants, and taxonomic assignment

Raw reads were trimmed and filtered using Fastp v0.20.1^66^, removing the first 10 nucleotides and discarding reads with one or more Ns. Next, we used a right-to-left sliding window method with a size of 4 to discard fragments with a Q-score lower than 30. High-quality reads were de-noised, filtered against chimeric PCR artifacts, and merged using the R package DADA2 v2.1.18^67^, producing clean 16S rRNA gene amplicon sequence variants (ASVs). The taxonomic assignment was performed with the same R package using SILVA database v138.1^68^ as reference. Finally, the taxonomic table and ASV reads count table were saved into a phyloseq v1.4 object for downstream analyses^44^. Alpha diversity measures from rarefied samples were performed through the phyloseq v1.4 R package. In this analysis, all samples were randomly subsampled to 90% of the sample with the lowest number of reads (106.724 reads). Rarefaction curves were generated using the rarecurve function from vegan v2.6.2^69^.

### Differential abundance of ASVs, pathway enrichment, and co-occurrence network analyses

The differential abundance of ASV was calculated using DESeq2 v2.1.36^70^ with default parameters. The comparisons were performed separately while considering the infected *versus* healthy samples from digesta and intestinal mucosa. To be considered differentially abundant, we used a p-value cutoff lower than or equal to 0.05. The inference of differentially enriched pathways was obtained using PICRUSt2 v2.4.2^71^ with default parameters; meanwhile, the statistics and visualization were generated using STAMP v2.1.3^72^ while considering a two-sided Welch’s t-test with the Benjamini–Hochberg correction and an adjusted p-value lower than or equal to 0.05. Finally, the co-occurrence network analysis was performed using cooccur v1.3^73^ (while considering a p-value lower than or equal to 0.05) and visualized using visNetwork v2.1 (https://datastorm-open.github.io/visNetwork/). The co-occurrence networks were further explored using NetShift v1^74^.

### Nonmetric multidimensional scaling (NMDS) analysis

We computed Bray–Curtis dissimilarity indexes of the bacterial network ASVs for each sample and plotted nonmetric multidimensional scaling between healthy and sick fish using a specialized R-package algorithm. The colored circles indicate sample clustering according to sample type. The digesta and gut mucosa of healthy fish are shown as red and green dots respectively. The digesta and gut mucosa of SRS-positive fish are shown in blue-green and purple, respectively. We used the adonis2 function from the vegan R package to calculate a Permutational Multivariate Analysis of Variance Using Distance Matrices (a PERMANOVA test) to test if the ASV profiles were significantly different for the four subject groups (healthy digesta, infected digesta, healthy intestinal mucosa and infected intestinal mucosa). We report these statistical calculations in an Excel file included in the Supplementary Information.

### Supplementary Information

Supplementary Information 1.

Supplementary Information 2.

## Data Availability

The datasets generated and analyzed during this investigation are available at the Sequence Read Archive (SRA) repository at the National Center for Biotechnology Information (NCBI) under the reference number PRJNA895462 and accessible at this link: https://www.ncbi.nlm.nih.gov/sra/PRJNA895462.
