# Publisher Correction: Bacterial networks in Atlantic salmon with Piscirickettsiosis

**DOI:** 10.1038/s41598-023-45850-5

**Published:** 2023-11-01

**Authors:** Yoandy Coca, Marcos Godoy, Juan Pablo Pontigo, Diego Caro, Vinicius Maracaja‑Coutinho, Raúl Arias‑Carrasco, Leonardo Rodríguez‑Córdova, Marco Montes de Oca, César Sáez‑Navarrete, Ian Burbulis

**Affiliations:** 1 Doctorado en Ciencias de la Ingeniería, Departamento de Ingeniería Química y Bioprocesos, Escuela de Ingeniería, Pontificia Universidad Católica de Chile, Avenida Vicuña Mackenna 4860, 7820436 Santiago, Región Metropolitana Chile; 2 Centro de Investigaciones Biológicas Aplicadas (CIBA), Avenida Lago Panguipulli 1390, Puerto Montt, Región de Los Lagos Chile; 3 Laboratorio Institucional, Facultad de Ciencias de la Naturaleza, Escuela de Medicina Veterinaria, Universidad San Sebastián, Sede Patagonia, Avenida Lago Panguipulli 1390, Puerto Montt, Región de Los Lagos Chile; 4 Centro de Modelamiento Molecular, Biofísica y Bioinformática (CM2B2), Facultad de Ciencias Químicas y Farmacéuticas, Universidad de Chile, Avenida Dr. Carlos Lorca Tobar 964, 8380494 Santiago, Región Metropolitana Chile; 5 Beagle Bioinformatics, Santiago, Región Metropolitana Chile; 6 Programa Institucional de Fomento a la Investigación, Desarrollo e Innovación (PIDi), Universidad Tecnológica Metropolitana, Avenida Dieciocho 161, 8330383 Santiago, Región Metropolitana Chile; 7 Facultad de Ingeniería, Escuela de Ingeniería, Universidad Santo Tomás, Avenida Ejército Libertador 146, Santiago, Región Metropolitana Chile; 8 Departamento de Ingeniería Química y Bioprocesos, Pontificia Universidad Católica de Chile, Avenida Vicuña Mackenna 4860, 7820436 Santiago, Región Metropolitana Chile; 9 Centro de Investigación en Nanotecnología y Materiales Avanzados (CIEN-UC), Pontificia Universidad Católica de Chile, Avenida Vicuña Mackenna 4860, 7820436 Santiago, Región Metropolitana Chile; 10 Centro de Investigación Biomédica, Facultad de Medicina y Ciencia, Universidad San Sebastián, Sede Patagonia, Avenida Lago Panguipulli 1390, Puerto Montt, Región de Los Lagos Chile; 11 Unidad de Genómica Avanzada, Facultad de Ciencias Químicas y Farmacéuticas, Universidad de Chile, Avenida Dr. Carlos Lorca Tobar 964, 8380494 Santiago, Región Metropolitana Chile

Correction to: *Scientific Reports* https://doi.org/10.1038/s41598-023-43345-x, published online 13 October 2023

In the original version of this Article, Affiliation 6 and 11 were not listed in the correct order. The correct affiliations are listed below:

^6^Programa Institucional de Fomento a la Investigación, Desarrollo e Innovación (PIDi), Universidad Tecnológica Metropolitana, Avenida Dieciocho 161, 8330383 Santiago, Región Metropolitana, Chile.

^7^Facultad de Ingeniería, Escuela de Ingeniería, Universidad Santo Tomás, Avenida Ejército Libertador 146, Santiago, Región Metropolitana, Chile.

^8^Departamento de Ingeniería Química y Bioprocesos, Pontificia Universidad Católica de Chile, Avenida Vicuña Mackenna 4860, 7820436 Santiago, Región Metropolitana, Chile.

^9^Centro de Investigación en Nanotecnología y Materiales Avanzados (CIEN-UC), Pontificia Universidad Católica de Chile, Avenida Vicuña Mackenna 4860, 7820436 Santiago, Región Metropolitana, Chile.

^10^Centro de Investigación Biomédica, Facultad de Medicina y Ciencia, Universidad San Sebastián, Sede Patagonia, Avenida Lago Panguipulli 1390, Puerto Montt, Región de Los Lagos, Chile.

^11^Unidad de Genómica Avanzada, Facultad de Ciencias Químicas y Farmacéuticas, Universidad de Chile, Avenida Dr. Carlos Lorca Tobar 964, 8380494 Santiago, Región Metropolitana, Chile.

The original Article has been corrected.

