# Supplementary material for: Bacterial networks in Atlantic salmon with Piscirickettsiosis
Source: Sci Rep. 2023 Oct 13;13:17321. doi: 10.1038/s41598-023-43345-x (PMC10576039; doi:10.1038/s41598-023-43345-x)
Supplement: Supplementary file 1 — Supplementary Information 1. [file 41598_2023_43345_MOESM1_ESM.pdf]

Bacterial networks in Atlantic salmon with Piscirickettsiosis

Authors:

Yoandy Coca [1,+]; Marcos Godoy [2,3,+]; Juan Pablo Pontigo [3]; Diego Caro [2]; Vinicius Maracaja-Coutinho [4, 5], Raúl Arias-Carrasco [6], Leonardo Rodríguez-Córdova [7], Marco Montes de Oca [2], César Sáez-Navarrete [8,9]; Ian Burbulis [10,*]

Supplementary Figures and Tables:

Supplementary Figure 1 | Histopathology of SRS.

Supplementary Figure 2 | Quantitative comparison of 16S rRNA gene amplicon abundance.

Supplementary Figure 3 | Rarefaction curve of 16S rRNA gene amplicons.

Supplementary Figure 4 | Observed statistics of ASV quality.

Supplementary Figure 5 | Relative abundance of bacterial taxa in healthy and SRS fish.

Supplementary Figure 6 | Relative abundance of various Atlantic salmon pathogens.

Supplementary Figure 7 | Principle component analysis of 16S rRNA gene amplicons.

Supplementary Table 1 | Description of sampling times and locations.

Supplementary Table 2 | List of bacterial families that co-occur with *Piscirickettsiaceae*.

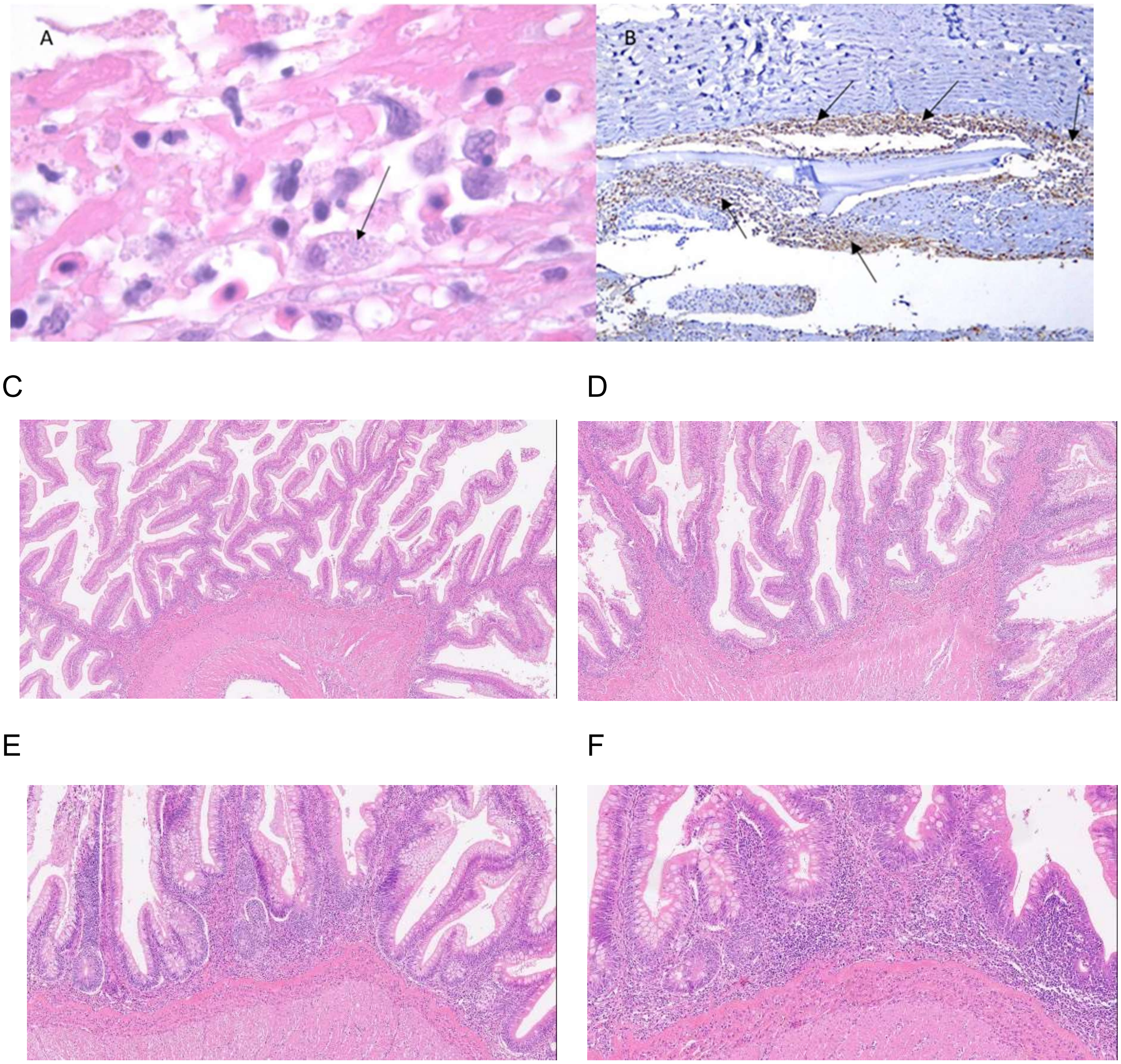

Supplementary Figure 1 | Histopathology of SRS. (**A**) Histopathology of *P. salmonis* infection in the skin of Atlantic salmon. These sections are representative of intracellular *P. salmonis*, indicated by the arrow. (**B**) Representative image of immuno-histochemical detection of *P. salmonis* in the skin of Atlantic salmon. Positive detection appears brown with DAB substrate. (**C + D**) Representative histopathology of healthy gut mucosa. (**E + F**) Representative histopathology of gut mucosal inflammation in Atlantic salmon with skin lesions positive for *P. salmonis*.

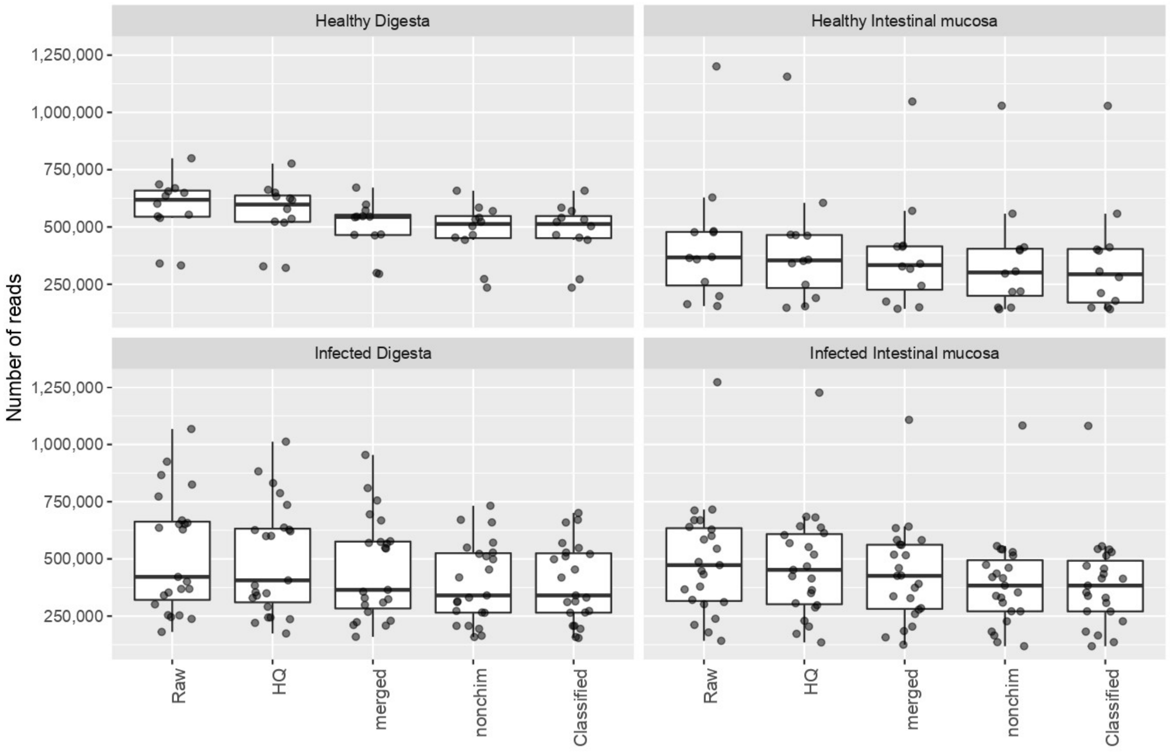

Supplementary Figure 2 | Quantitative comparison of 16S amplicon abundance. Reads count of 16S amplicon samples analyzed in healthy digesta, healthy intestinal mucosa, infected digesta, and infected intestinal mucosa. From the R package: The lower and upper box borders correspond to the first and third quartiles (the 25th and 75th percentiles). The upper whisker extends from the hinge to the largest value no further than 1.5 * IQR from the hinge (where IQR is the inter-quartile range, or distance between the first and third quartiles). The lower whisker extends from the hinge to the smallest value at most 1.5 * IQR of the hinge.

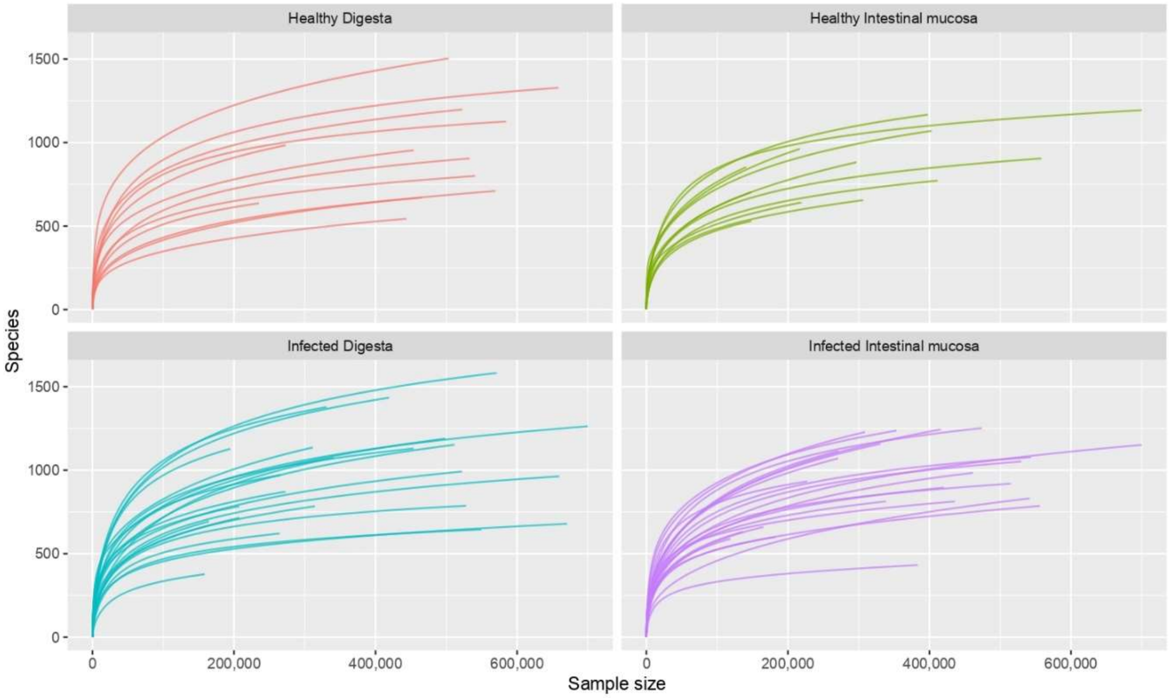

Supplementary Figure 3 | Rarefaction curves of 16S amplicons. The cleaned samples data was analyzed in healthy digesta, healthy intestinal mucosa, infected digesta, and infected intestinal mucosa.

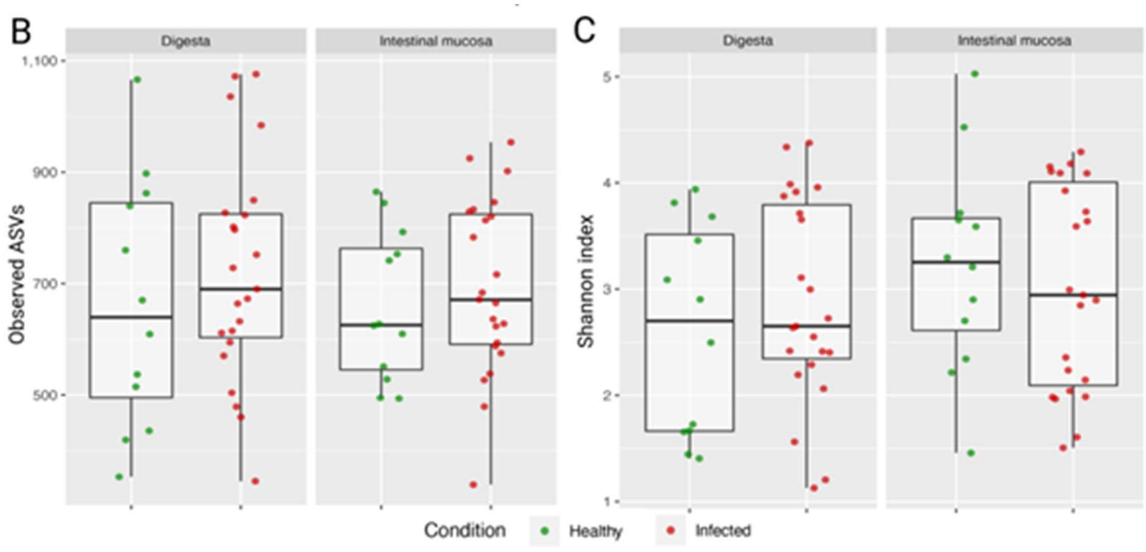

Supplementary Figure 4 | Observed statistics of ASV quality. **(B)** Observed richness of ASVs. **(C)** Shannon index of bacterial alpha diversity estimation in both digesta and intestinal microbiota of salmon with and without clinical SRS. From the R package: The lower and upper box borders correspond to the first and third quartiles (the 25th and 75th percentiles). The upper whisker extends from the hinge to the largest value no further than 1.5 * IQR from the hinge (where IQR is the inter-quartile range, or distance between the first and third quartiles). The lower whisker extends from the hinge to the smallest value at most 1.5 * IQR of the hinge. Data points representing healthy and sick fish are shown in green and red, respectively.

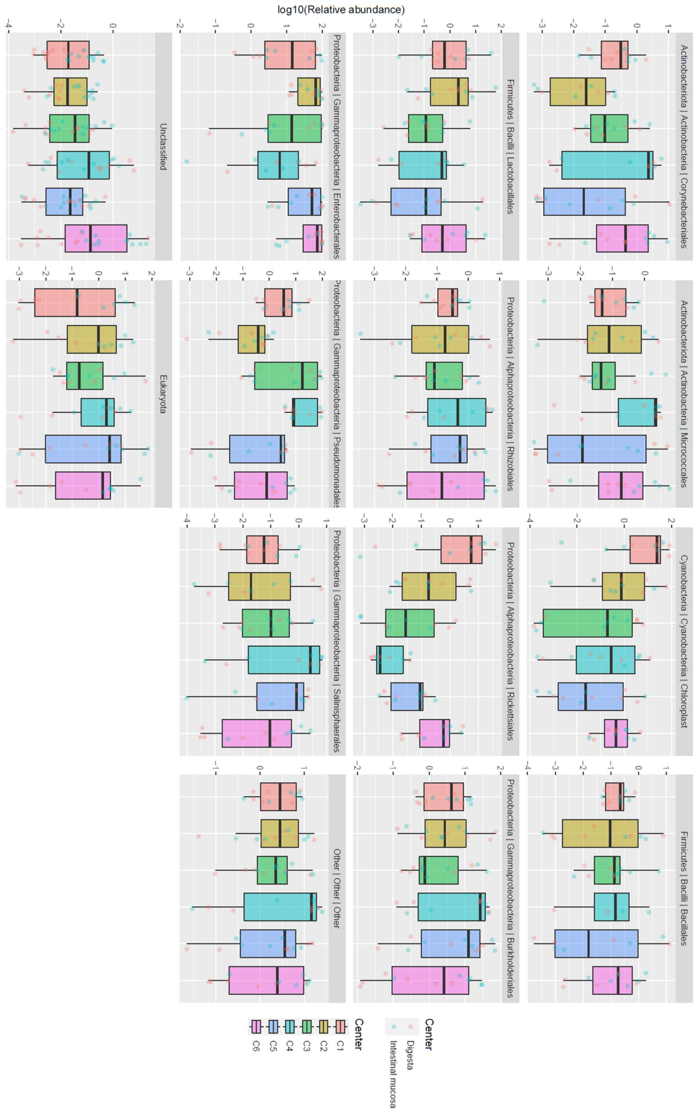

Supplementary figure 5 | Relative abundance of bacterial taxa in healthy and SRS fish. The farm sites where fish samples were collected are color coded (Center, C1, C2, etc.). Each phylum is indicated at the top of each box. The $\log_{10}$ abundance of specific phyla are indicated along the X-axis of each box. Individual fish samples are indicated as dots. The dots corresponding to the digesta and gut mucosa are shown as pink and blue-green, respectively. The lower and upper box borders correspond to the first and third quartiles (the 25th and 75th percentiles). The upper whisker extends from the hinge to the largest value no further than 1.5 * IQR from the hinge (where IQR is the inter-quartile range, or distance between the first and third quartiles). The lower whisker extends from the hinge to the smallest value at most 1.5 * IQR of the hinge.

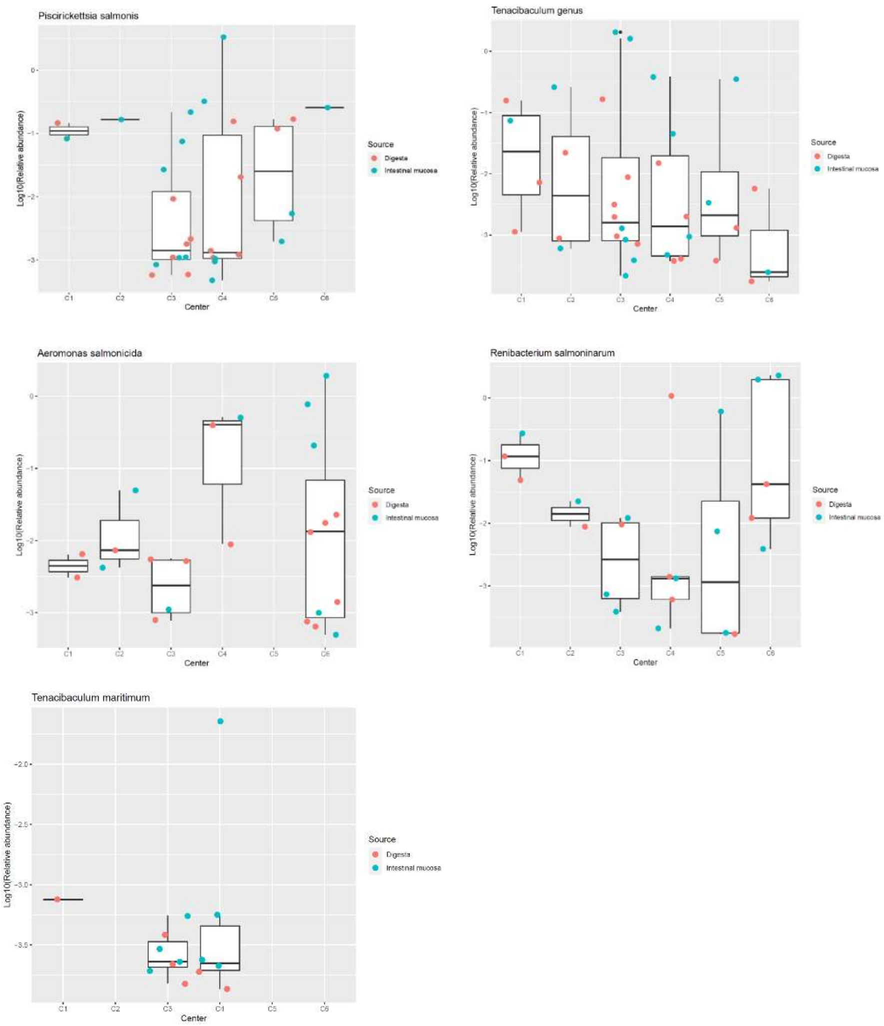

Supplementary Figure 6 | Relative abundance of Atlantic salmon pathogens. The relative abundance of ASVs corresponding to other common bacterial pathogens of Atlantic salmon present in the sequencing data. The $\log_{10}$ of the relative abundance of each pathogen is graphed on the Y axis. The farm site from where specific fish samples were collected is marked along the X-axis. Each dot represents a positive detection in one of the fish samples at that site. The dots representing digesta and intestinal mucosa are color coded pink and blue-green, respectively. The lower and upper box borders correspond to the first and third quartiles (the 25th and 75th percentiles). The upper whisker extends from the hinge to the largest value no further than 1.5 * IQR from the hinge. The lower whisker extends from the hinge to the smallest value at most 1.5 * IQR of the hinge.

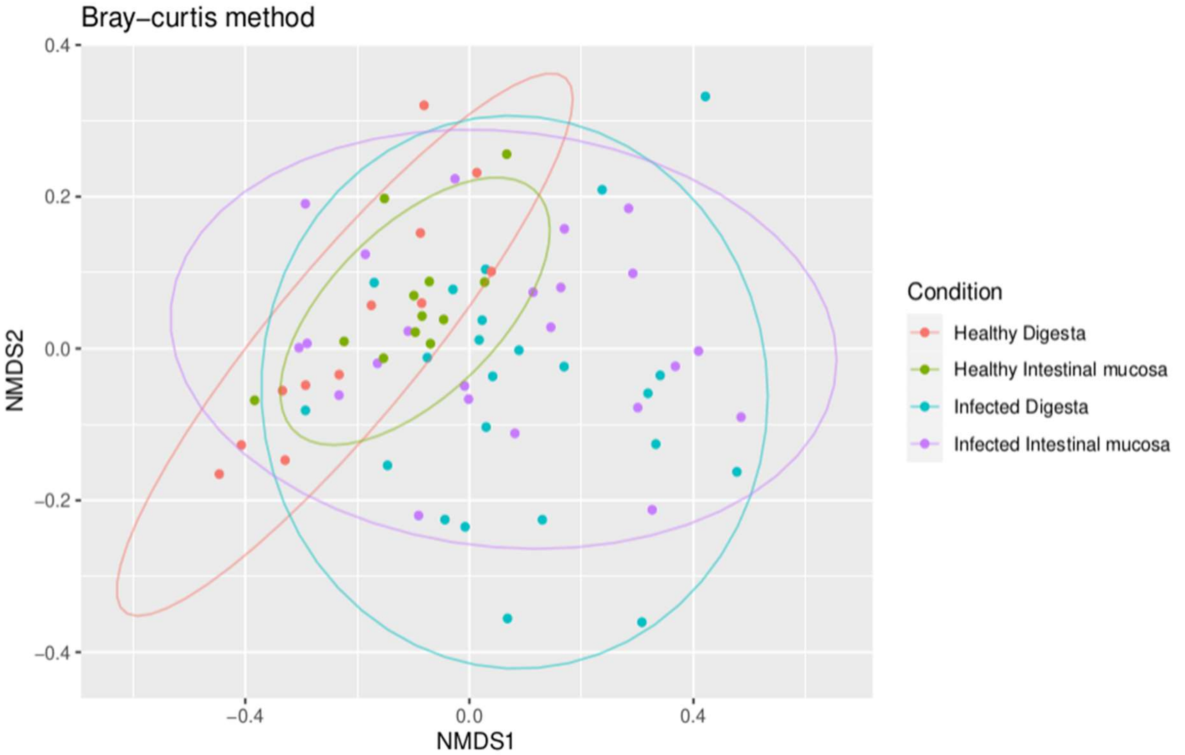

Supplementary Figure 7 | Nonmetric multidimensional scaling (NMDS) plot of microbial 16S rRNA gene profile dissimilarities. The beta diversity of the 16S amplicon samples was calculated according to the Bray–Curtis method. The healthy digesta and gut mucosa are indicated as red and green dots respectively. The digesta and gut mucosa from SRS-positive fish are shown in blue-green and purple, respectively.

Healthy Condition	Sampling Data	Salmonid Farm	Sample Type			
			Digesta		Intestinal mucosa	
			Number of samples	Number of samples	Number of samples	Number of samples
Salmonids Infected with SRS	January and February	C1	6	6	6	6
		C2	6	6	6	6
		C3	6	6	6	6
		C4	5	5	5	5
Total Infected Salmonids			23	23	23	23
Healthy Salmonids	January and February	C5	6	6	6	6
		C6	6	6	6	6
Total Healthy Salmonids			12	12	12	12

Supplementary Table 1 | Description of sampling times and locations. Sampling system was executed in the farming centers of the Los Lagos and Aysén Regions (Chile) during infectious outbreaks of Salmonid Rickettsial Septicemia (SRS).

Negative		Positive	
**Family**	**P-value**	**Family**	**P-value**
Enterococcaceae	0.00019	*Omnitrophaceae*	0
Idiomarinaceae	0.00023	*Arcobacteraceae*	0.00001
Clostridiaceae	0.00057	*Porticoccaceae*	0.00898
Peptostreptococcaceae	0.0008	*Hydrogenophilaceae*	0.01133
Alcaligenaceae	0.00163	*Flavobacteriaceae*	0.01687
Mycoplasmataceae	0.00217	*PRD18C08*	0.01891
Pasteurellaceae	0.00377	*Rhodocyclaceae*	0.02247
Caldatribacteriaceae	0.00488	*Pseudoalteromonadaceae*	0.02752
Streptococcaceae	0.00548	*Methanobacteriaceae*	0.03876
Planococcaceae	0.01034	*Azospirillaceae*	0.03876
Erwiniaceae	0.01117		
Family XI	0.01117		
Aneurinibacillaceae	0.01192		
Dermabacteraceae	0.01192		
Spirosomaceae	0.01485		
Lachnospiraceae	0.01584		
Carnobacteriaceae	0.01724		
Rhodobacteraceae	0.01891		
Lactobacillaceae	0.02974		
Limnotrichaceae	0.03655		
Dongiaceae	0.03668		
Hungateiclostridiaceae	0.03668		
Solimonadaceae	0.03668		
Sporolactobacillaceae	0.03668		
Unknown Family	0.03668		
Vagococcaceae	0.04562		
Selenomonadaceae	0.04626		

Supplementary Table 2 | List of bacterial families that co-occur with *Piscirickettsiaceae*. Statistically significant co-occurrence between other families and *Piscirickettsiaceae* according to the PICRUSt2 tool.
